# Predicting plant biomass accumulation from image-derived parameters

**DOI:** 10.1093/gigascience/giy001

**Published:** 2018-01-16

**Authors:** Dijun Chen, Rongli Shi, Jean-Michel Pape, Kerstin Neumann, Daniel Arend, Andreas Graner, Ming Chen, Christian Klukas

**Affiliations:** 1 Leibniz Institute of Plant Genetics and Crop Plant Research (IPK), Corrensstrasse 3, 06466 Gatersleben, Germany; 2 Department of Bioinformatics, College of Life Sciences, Zhejiang University, Hangzhou 310058, China; 3 Present address: Department for Plant Cell and Molecular Biology, Institute for Biology, Humboldt-Universität zu Berlin, 10115 Berlin, Germany; 4 Present address: Digitalization in Research and Development (ROM), BASF SE, 67056 Ludwigshafen am Rhein, Germany

**Keywords:** barley, high-throughput phenotyping, phenomics, biomass, modeling

## Abstract

**Background:**

Image-based high-throughput phenotyping technologies have been rapidly developed in plant science recently, and they provide a great potential to gain more valuable information than traditionally destructive methods. Predicting plant biomass is regarded as a key purpose for plant breeders and ecologists. However, it is a great challenge to find a predictive biomass model across experiments.

**Results:**

In the present study, we constructed 4 predictive models to examine the quantitative relationship between image-based features and plant biomass accumulation. Our methodology has been applied to 3 consecutive barley (*Hordeum vulgare*) experiments with control and stress treatments. The results proved that plant biomass can be accurately predicted from image-based parameters using a random forest model. The high prediction accuracy based on this model will contribute to relieving the phenotyping bottleneck in biomass measurement in breeding applications. The prediction performance is still relatively high across experiments under similar conditions. The relative contribution of individual features for predicting biomass was further quantified, revealing new insights into the phenotypic determinants of the plant biomass outcome. Furthermore, methods could also be used to determine the most important image-based features related to plant biomass accumulation, which would be promising for subsequent genetic mapping to uncover the genetic basis of biomass.

**Conclusions:**

We have developed quantitative models to accurately predict plant biomass accumulation from image data. We anticipate that the analysis results will be useful to advance our views of the phenotypic determinants of plant biomass outcome, and the statistical methods can be broadly used for other plant species.

## Introduction

Biomass accumulation is an important indicator of crop final product and plant performance. It is thus considered a key trait in plant breeding, agriculture improvement, and ecological applications. The conventional approach of measuring plant biomass is very time-consuming and labor-intensive as plants need to be harvested destructively to obtain the fresh or dry weight [1]. Moreover, the destructive method makes multiple measurements of the same plant over time impossible. With the development of new technology, digital image analysis has been used more broadly in many fields, as well as in plant research [2–4]. It allows faster and more accurate plant phenotyping and has been proposed as an alternative way to infer plant biomass [2, 3, 5].

In recent years, plant biomass has been subject to intensive investigation by using high-throughput phenotyping (HTP) approaches in both controlled growth chambers [2, 3, 6–11] and field environments [5, 12–17], demonstrating that the ability of imaging-based methods to infer plant biomass accumulation. For example, significant genotypic and environmental effects on plant biomass in Setaria were revealed by the Bellwether Phenotyping Platform in a controlled environmental condition [10]. Yang et al. [11] showed that predicted rice biomass (including shoot fresh and dry weight) based on image-derived morphological and texture features provided a relatively more complete representation than manual measurements in dissecting its genetic architecture. In this regard, optimized models plus image-derived features from HTP systems will improve the power of dissecting genetic architecture of complex traits. Although there are some developed models for predicting plant biomass, most of them have certain limitations. For example, Golzarian et al. (2011) modeled the plant biomass (dry weight) in wheat (*Triticum aestivum* L.) as a linear function of projected area, assuming plant density was constant. However, this method underestimated the dry weight of salt-stressed plants and overestimated that of control plants. Even though the authors argued that the bias was largely related to plant age and the model might be improved by including the factor of plant age [3], the differences in plant density between stressed and control plants may have been caused by different physiological properties of plants rather than plant age. In another study, Busemeyer et al. (2013) developed a calibrated biomass determination model for triticale (x *Triticosecale* Wittmack L.) under field conditions based on multiple linear regression analysis of a diverse set of parameters, considering both the volume of the plants and their density. Indeed, this model largely improved the prediction accuracy of the calibration models based on a single type of parameters and can precisely predict biomass accumulation across environments [15]. However, Buesmeyer et al. (2013) used very limited traits for the model and question whether it could be applied broadly in other cases. As mentioned by Yang et al*.* (2014), noticeable improvement was achieved by adding morphological features or texture features to the biomass-predicting model [11]. This suggests that adding more information/traits could improve the predictive performance of models. Therefore, a more effective and powerful model is needed to overcome these limitations and to allow better utilization of the image-based plant features, which are obtained from noninvasive phenotyping approaches.

Individual studies have recently shown that the prediction accuracy of plant biomass based on image-derived features is relatively high even using the simplest linear regression models [3, 10, 18]. However, the performance of nonlinear predictive models has not been well evaluated. Further, it is still challenging to apply these models across experiments that are performed in different environmental conditions or with different treatments due to a lack of datasets for assessment. In this study, we present a general framework for investigating the relationships between plant biomass (referred to as shoot biomass hereafter) and image-derived parameters. We applied a multitude of supervised and unsupervised statistical methods to investigate different aspects of biomass determinants by a list of representative phenotypic traits in 3 consecutive experiments in barley. The results showed that image-based features can accurately predict plant biomass output and collectively reflect large proportions of the variation in biomass accumulation. We elucidated the relative importance of different feature categories and of individual features in the prediction of biomass accumulation. The differences in the contribution of the image-based features for the prediction of the 2 types of biomass measurements, fresh weight and dry weight, were compared as well. Furthermore, our models were tested for the possibility of predicting plant biomass in different experiments with different treatments.

## Results

### Development of statistical models for modeling plant biomass accumulation using image-based features

**Figure 1:**
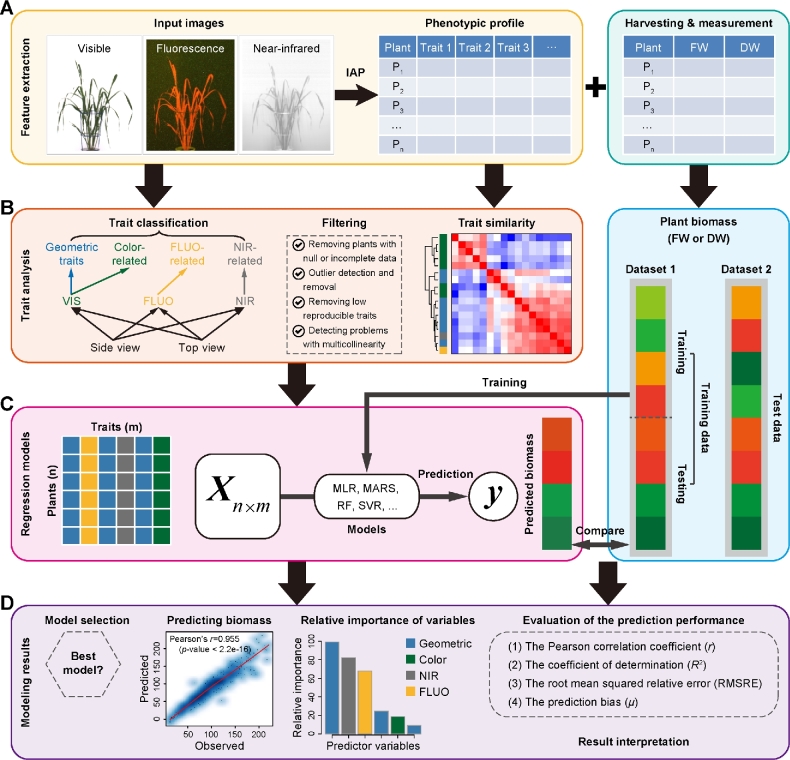
Modeling pipeline for predicting plant biomass accumulation based on image-derived parameters. A, Input data, including high-throughput image data and manually measured biomass data. Plants were phenotyped using various cameras such as visible (or color), fluorescence, and near-infrared sensors. Image analysis was performed with IAP software [10] for feature extraction. The same plants were harvested and measured at the end of growth. Generally, 2 types of biomass were measured: fresh weight and dry weight. B, Trait processing. All the phenotypic traits were grouped into 4 categories: geometric, color-related, FLUO-related, and NIR-related traits. Phenotypic data were subjected to quality check to remove low-quality data. C, Each plant was described by a list of traits, resulting in a predictor matrix whose rows represent plants and columns represent image-based traits. This matrix was used to predict plant biomass accumulation by MLR, MARS, RF, and SVR models. The right panel represents the schema of model validation. In the first schema, a dataset (Dataset 1) was divided into training set and testing set in a 10-fold cross-validation manner. In the second schema, the whole of 1 dataset (Dataset 1) was used for training and another dataset (Dataset 2) was used for testing. D, Model selection, evaluation, and result interpretation. The correlation of the predicted values and measured values was used to assess the overall performance of the model.

In the previous studies [19, 20], we have shown that a single phenotypic trait—3-dimensional digital volume, which is a derived feature from projected side and top areas—can be reasonably predictive to estimate plant biomass accumulation. We expect that the predictive power could be improved when multiple phenotypic traits are combined in a prediction model as plant biomass is determined not only by structural features but also by density (physiological properties). To further investigate the relationship between image-derived parameters and plant biomass accumulation, deep phenotyping data that contain both structural (e.g., geometric traits) and physiological traits (e.g., plant moisture content as reflected by near-infrared [NIR]-related traits) were analyzed (Fig. 1A and B). Pot weights of the plants were not included for the analysis, although they were weighed regularly. It might reflect the growth tendency of the whole plant (shoots and roots), where herein we focused mainly on shoots.

Models were constructed to quantify the ability of imaging-based features to statistically predict the biomass accumulation. The models were developed by using 4 widely used machine-learning methods (Fig. 1C): multivariate linear regression (MLR), multivariate adaptive regression splines (MARS), random forest (RF), and support vector regression (SVR), which have extensively been used in accurate prediction of gene expression [21–25] and DNA methylation levels [26–29]. We combined the biomass measurements (fresh weight [FW] and/or dry weight [DW]) with image-based features and then divided them into a training dataset and a test dataset. A model was trained on the training dataset and has then been applied to the test dataset to predict the plant biomass. The relationship between plant biomass accumulation and image-based features was assessed based on the criterion of the Pearson correlation coefficient (*r*) between the predicted values and the actual values, or the coefficient of determination (*R*^2^; the percentage of variance of biomass explained by the model) (Fig. 1D).

**Figure 2:**
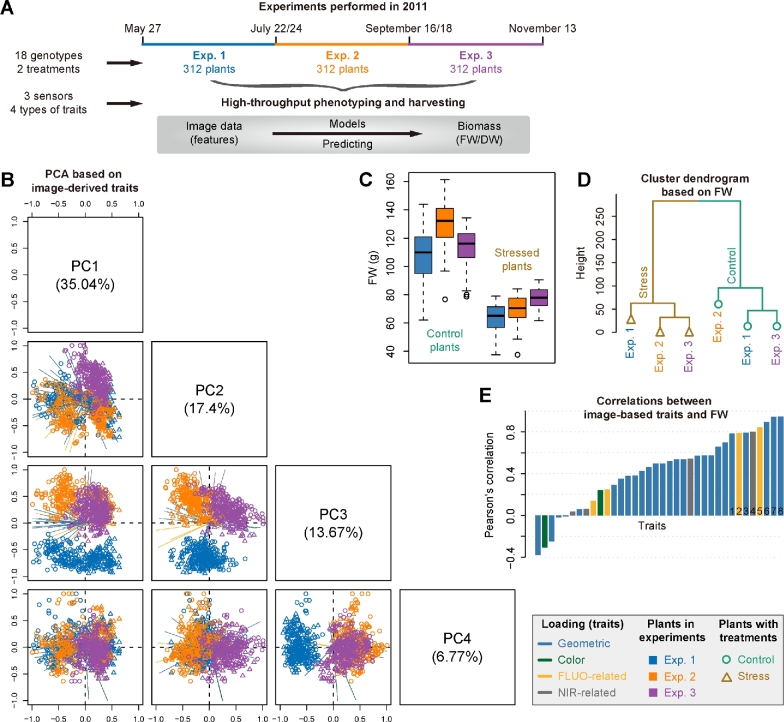
Predictability of image-based traits to plant biomass. A, Schema depicting 3 consecutive high-throughput phenotyping experiments in barley. Plants in each experiment were harvested for biomass measurements: fresh weight (for all experiments) and dry weight (only for experiment 1). B, Scatter plots showing projections of the top 4 PCs based on PCA of image-based data. The component scores (shown in points) are colored and shaped according to the experiments (as legend listed in the box). The component loading vectors (represented in lines) of all traits (as colored according to their categories) were superimposed proportionally to their contribution. C, Boxplot showing the distribution of FW across different experiments. D, A dendrogram from cluster analysis based on the means of FW data over genotypes. E, Pearson's correlation (mean values in the 3 datasets) between image-based traits and FW. Traits with the largest mean correlations values are labeled: 1: sum of leaf length (side view); 2: sum of FLUO intensity (side); 3: plant area border length (side); 4: sum of NIR intensity (top); 5: sum of FLUO intensity (top); 6: projected area (top); 7: projected area (side); and 8: digital volume.

Our methodology was applied to 3 consecutive experiments (Fig. 2A; Supplemental Table S1 and Data S1), which were designed to investigate vegetative biomass accumulation in response to 2 different watering regimes under semicontrolled greenhouse conditions in a core set of barley cultivars by noninvasive phenotyping [20, 30]. There were 312 plants with 18 genotypes for each experiment. Plants were monitored using 3 types of sensors (visible, fluorescence [FLUO], and NIR) in a LemnaTec-Scanalyzer 3D imaging system. An extensive list of phenotypic traits ranging from geometric (shape descriptors) to physiological properties (i.e., color-, FLUO-, and NIR-related traits) could be extracted from the image data (Supplemental Data S1) using our image processing pipeline IAP [19]. A representative list of traits for each plant in the last growth day were selected to test their ability to predict plant biomass.

### Coordinated patterns of plant image–based profiles and their relation to plant biomass

We extracted a list of representative and nonredundant phenotypic traits for each plant from image datasets for each experiment (see Materials and Methods) (Fig. 1B). In common for these experiments, overall 36 high-quality traits that describe plant growth status in the last growth day were obtained. As a result, each dataset was assigned a matrix whose elements were the signals of different features in different plants (Fig. 1C). Principal component analysis (PCA) (Fig. 2B) was applied to these datasets. We found that plants from different experiments with different treatments showed clearly distinct patterns of phenotypic profiles. For instance, stressed plants and control plants were separated using PCA by their first principal component (PC1) and also by the top clusters obtained in hierarchical clustering analysis (HCA), while plants from different experiments were distinguished by PC2 and PC3 in PCA or subordinate clusters in HCA. Accordingly, it could be observed that the biomass (e.g., FW) of plants from different experiments with different treatments was significantly different (2-way ANOVA, *P* < 2e-16) (Fig. 2C). The relationship was reflected by a dendrogram from cluster analysis based on the means of FW over genotypes (Fig. 2D). Furthermore, the overall phenotypic patterns of these plants were similar to their biomass output (Fig. 2B–D), revealing that these image-based features were potential factors reflecting the accumulation of plant biomass. We thus explored the relationship between the signals of these image-based features and the level of plant biomass output. We calculated the correlation coefficients for each dataset. The correlation patterns were consistent for different datasets, and more than half of the features revealed high correlation coefficients (*r* > 0.5) (Fig. 2E). Interestingly, both structural features (such as digital volume, projected area, and the length of the projected plant area border) and density-related features (such as NIR and FLUO intensities) were involved in the top ranked features.

### Relating image-based signals to plant biomass output

The above analyses suggest that plant biomass can be at least partially inferred from image-based features. To examine which model has the best performance and to select an appropriate model for biomass prediction, we then applied our regression models (Fig. 1C) to predict plant biomass using image-based features. Our analyses were focused on the first experiment (i.e., Exp 1), as the phenotypic traits of the corresponding dataset have been intensively investigated in our previous study [20]. In this experiment, plant biomass was quantified in 2 forms: FW and DW. We selected a collection of 45 image-derived parameters from this dataset that were nonredundant and highly representative.

**Figure 3:**
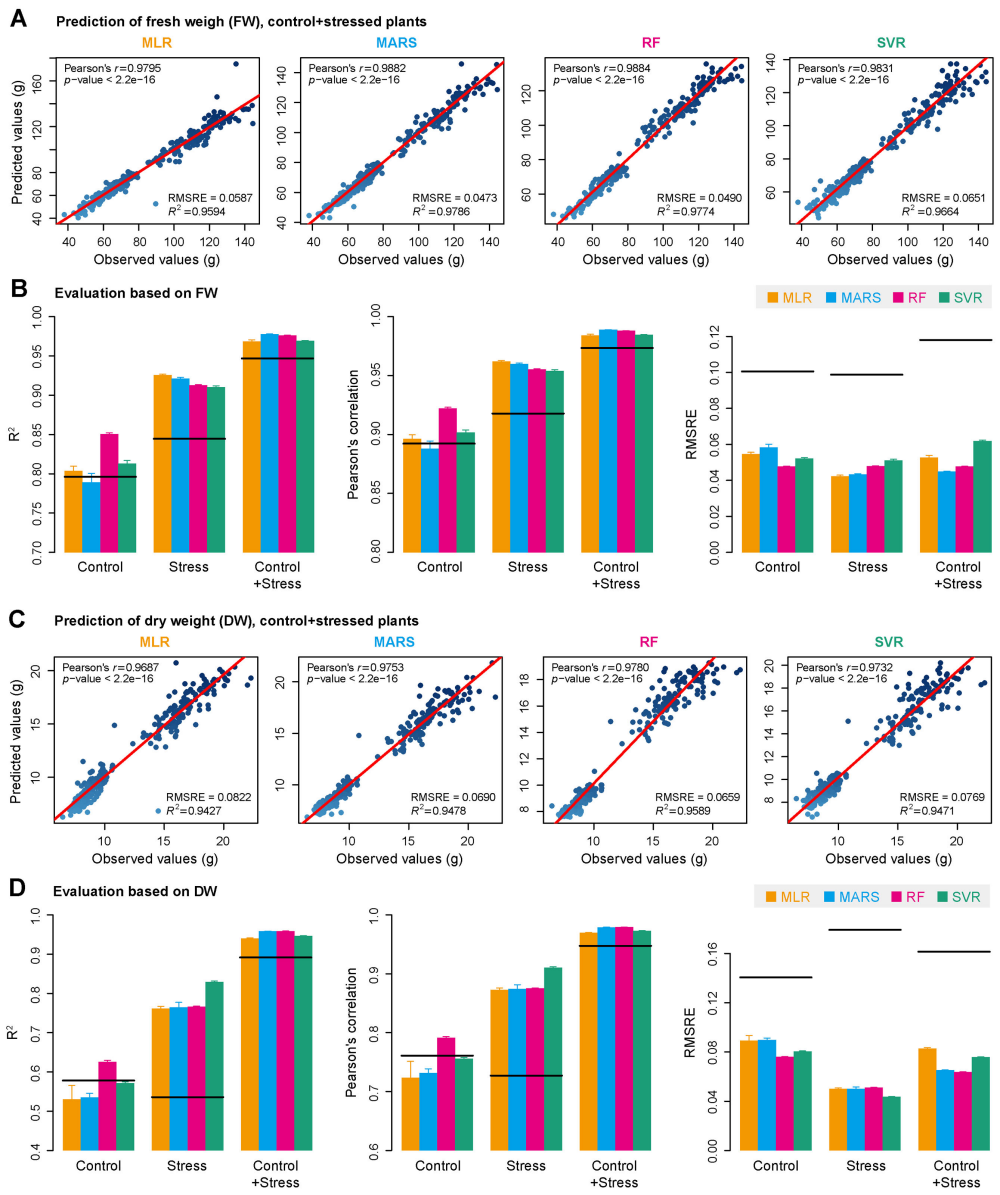
Quantitative relationship between image-based features and plant biomass. A and C, Scatter plots of manually measured plant biomass (fresh weight and dry weight) vs predicted biomass values using 4 prediction models: MLR, MARS, RF, and SVR. The red line indicates the expected prediction (*y* = *x*). The quantitative relationship between image-based features and biomass was evaluated by Pearson's correlation coefficient (PCC *r* and its corresponding *P*-value), RMSRE, and the percentage of variance explained by the models (the coefficient of determination *R*^2^). B and D, Summary of the predictive power of each regression model. The results were based on 10-fold cross-validation with 10 trials. Models were evaluated based on control plants, stressed plants, and the whole set of plants. The solid lines represent the predictive performance based on the single “digital volume” feature.

We next tried to predict FW and DW based on this set of image-derived features using 4 different regression models (MLR, RF, SVR, and MARS) (Fig. 3). The models were tested on control plants, stressed plants, and the whole set of plants, respectively (Fig. 3A and C). The prediction accuracy of our models (the correlation coefficients between the predicted biomass and the actual biomass) was first compared with the ability of individual features to predict biomass. It was found that our models generally showed better prediction power than the single digital volume-based prediction (Fig. 3B and D), indicating that additional features improved the predictive power. Then the performance of these models was compared and evaluated. Overall, the performance of all the tested models was roughly similar for the prediction of both FW (Fig. 3B) and DW (Fig. 3D) under stressed conditions. The prediction accuracy of our models is still comparable to the results from previous studies [3, 6, 18] based on MLR models, even though many more features were considered in our study. The RF model slightly outperformed other models in predicting biomass of control plants, accounting for the most variance ( *R*^2^ =  0.85 for FW and *R*^2^ =  0.62 for DW) (Fig. 3B and D, left panels) and showed the best prediction accuracy (Pearson's correlation *r* = 0.93 for FW and *r* = 0.80 for DW) (Fig. 3B and D, middle panels). Of note, RF is the only model showing better performance than single digital volume-based prediction (Fig. 3D). In this study, we focused on the results from the RF method in the rest of analysis, although results from different methods were highly consistent and led to the same conclusions.

### Relative importance of different image-based features for predicting plant biomass

**Figure 4:**
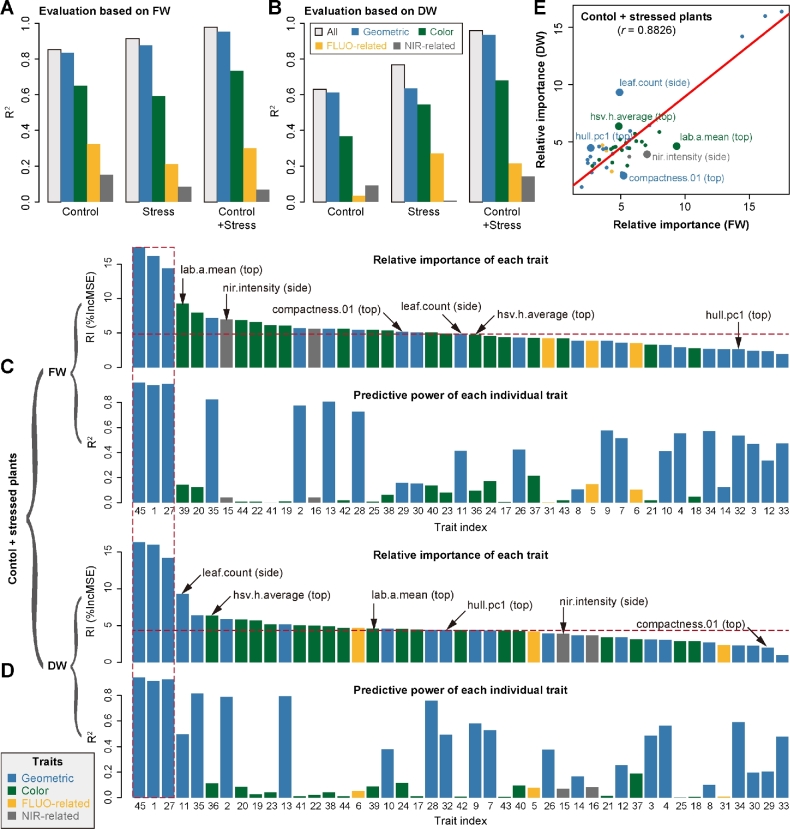
The relative importance of image-based features in the prediction of plant biomass. The capabilities of different types of image-based features to predict plant biomass based on evaluation of either fresh weight (A) or dry weight (B). The overall predictive accuracies of each type of features are indicated. Grey bars denote the predictive accuracy using all features. The relative importance of each feature in the RF model (upper panel) and the predictive accuracy of each individual feature as the single predictor (lower panel) based on investigation of either FW (C) or DW (D). The calculation was based on the whole set of plants (control and stressed plants). Note that feature labels are shared in the upper and lower panels. Features are shown in numbers as ordered by their names. The 3 features highlighted in the red dash box are digital volume, projected side area, and projected top area. E, Comparison of the relative importance of features in prediction of FW and DW. The top 6 most different features are highlighted and labeled.

As mentioned above, the image-based features could be classified broadly into 4 categories: plant structure properties, color-related features, NIR signals, and FLUO-based traits (Fig. 1B). The last 3 types of features reflect plant physiological properties and can be considered plant density–related traits and are thus related to their fresh or dry matter content. For each individual feature or each type of features, we constructed a degenerate model for biomass prediction using the corresponding feature(s) as the predictor(s). We compared the capability of each individual or type of feature for predicting biomass accumulation in the first experiment (i.e., experiment 1). Geometric features showed the most predictive power among the 4 categories for prediction of both FW and DW, but were slightly less predictive than all features in a full model (Fig. 4A and B). Strikingly, the predictability of other types of features (such as color-related and FLUO-based traits) was substantial, indicating that these traits may act as unforeseen factors in biomass prediction. In addition, the NIR-based features showed higher predictive capability for FW than for DW in control and stressed plants, revealing that NIR signals were important factors in determining FW accumulation.

Next, we investigated the relative importance (RI) of each feature for predicting biomass using a full model in the whole set of plants (i.e., “control + stressed plants”) (Fig. 4C and D, upper panels). In an RF model, the RI of a feature is calculated as the increase of prediction error (%IncMSE) when phenotypic data for this feature are permuted [31], and thus indicates the contribution of the feature after considering its intercorrelation in a model. We found that the top 10 most important features in the full model for predicting FW and DW included both structure- and density-related traits. As expected, projected area (from side or top view) and digital volume were the top ranked features, which have individually been considered proxies of shoot biomass in previous studies [3, 20, 18, 32–37]. However, several geometric and color-related features that are top ranked in the prediction have not been used in biomass predictions in previous analysis, although they are widely available among phenotyping platforms.

In principle, we would expect that highly important features in the full model would be related to a high predictive power in a degenerate model. Surprisingly, there was no clear correlation observed between the feature importance and its predictive power (Fig. 4C and D). For example, several color-related and NIR-based features that were in the top 10 list of the most important features revealed insubstantial predictive power in individual models. This observation implies that the relation of the underlying biomass determinants is extremely complex and not a linear combination of the investigated features.

Furthermore, we compared the relative importance of each feature in predicting FW and DW (Fig. 4E). Although a positive correlation (*r* = 0.88) between the feature importance for FW and DW could be observed, several features showed large differences in their ability to interpret FW or DW, including “nir.intensity” (derived from side view images), “compactness.01” (top), “hull.pc1” (top), “leaf.count” (side), “hsv.h.average” (top), and “lab.a.mean” (top). For instance, NIR intensity and plant compactness (top view) may be important for predicting FW but not for DW. We also performed the above analyses by using only control (Supplemental Fig. S1) or stressed plants (Supplemental Fig. S2), respectively. We found that the patterns of feature importance were distinct between these 2 groups of plants. For example, NIR intensity was ranked as a top 5 feature for predicting FW for stressed plants but was not substantially important for control plants. These findings suggest that there are differences in underlying plant biomass determinants in these kinds of treatment situations that are also reflected by their image-based phenotypic traits.

### Image-based features are predictive of plant biomass across experiments with similar conditions or treatments

**Figure 5:**
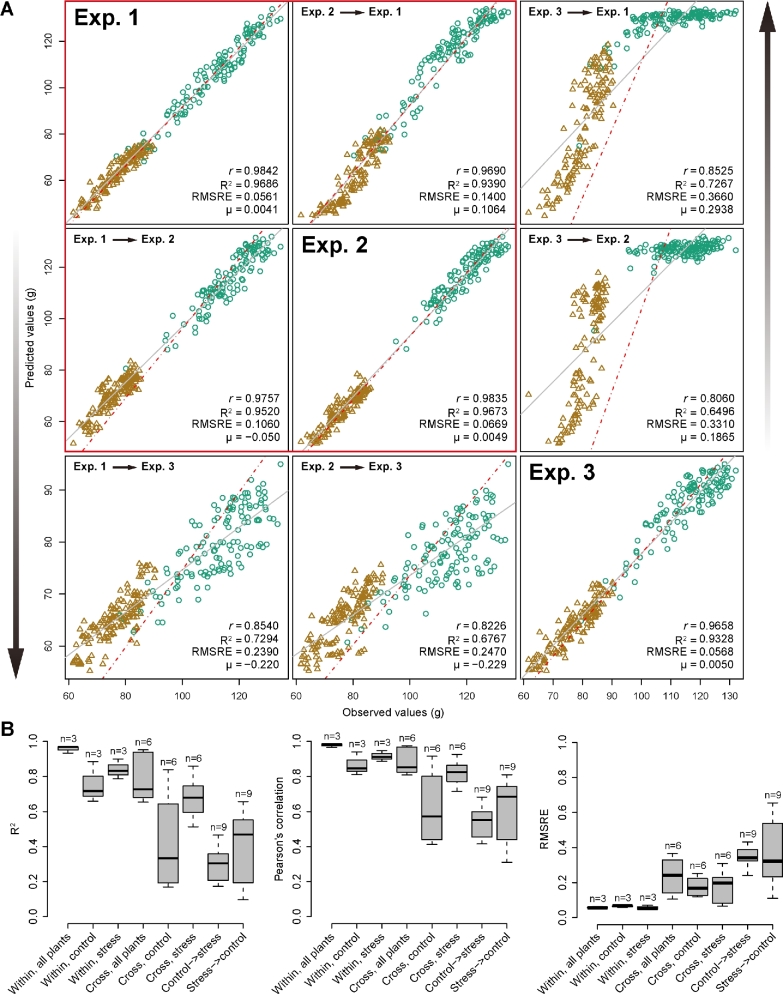
Comparison of prediction accuracy across different experiments. A, Biomass prediction across experiments. Models were trained using data from 1 experiment and were applied to another experiment for prediction. The whole set of plants (i.e., “control + stressed” plant) was used in the analysis. Brown triangles denote stressed plants, and green circles control plants. The red box indicates that the prediction accuracy is relatively high between experiments 1 and 2. B, Boxplots of coefficient determination (*R*^2^, left), Pearson's correlation coefficients (*r*, middle), and the root mean squared relative error (right) for different comparisons. “Within” denotes a model trained and tested on data from the same dataset with specific treatments (control, stress, or both), and “Cross” represents a model trained on 1 dataset and tested on another dataset. “Control → stress” denotes a model trained on data with control treatment and tested on data with stress treatment, and vice versa for “stress → control.” The number of possible analyses for each category is shown above the boxes.

In order to explore whether our models were generalizable across different experiments, we applied our models trained in 1 experiment to predict biomass (herein FW) in other experiments using a common set of features. Examples of such cross-experiment predictions are shown in Fig. 5A. We tested and illustrated all possibilities for cross-prediction using the whole set of plants in the corresponding experiment. In general, the prediction accuracy within individual experiments remained high (*r* > 0.97 and *R*^2^ >  0.93 for all 3 experiments) (Fig. 5B), revealing that our models were effectively predicting plant biomass based on image-derived feature signals among different experiments. Moreover, the prediction accuracy for cross-experiment prediction, especially between the first 2 experiments (*r* > 0.97 and *R*^2^ >  0.94), was still relatively high, implying that our models generally captured the relationships among the various image-based features. However, the third experiment had relatively weaker correlations than the other 2 experiments for predicting biomass (with *r* > 0.81 and *R*^2^ >  0.65) (Fig. 5A). This might be mainly due to seasonal (temperature and illumination) differences that caused different plants behaviors, namely lower biomass for both control and stressed plants in experiment 3 [30]. This suggests that different plant growth conditions might cause some variation for cross-experiment prediction.

**Figure 6:**
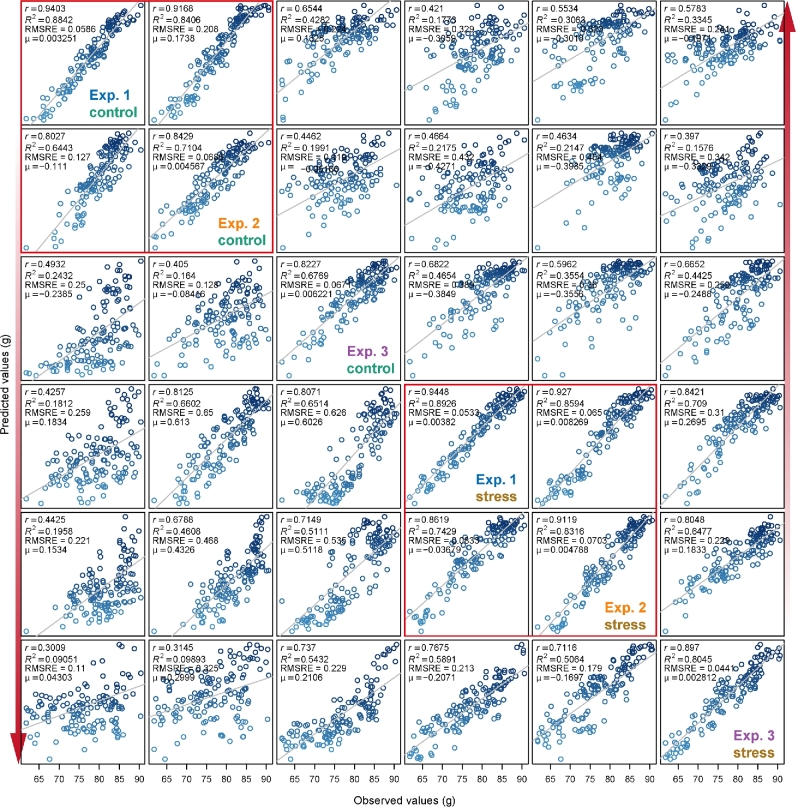
Comparison of prediction accuracy across different treatments. Refer to Fig. 5A for legend. The analysis was performed for control and stressed plants separately.

At the same time, we tested cross-predictability of our models using treatment-specific data in the experiments (Fig. 6). Similar results were obtained as above using the whole dataset (Fig. 5B). The weak predictive power for cross-prediction involving control plants from the third experiment was most clearly observable in the low accuracy in the biomass prediction of this particular subset of plants. Generally, control and stressed plants were found to have very weak predictive power when related to each other (Fig. 6), as also supported by the distinct patterns of relative feature importance between these 2 plant groups (Supplemental Figs S1 and S2). For each experiment, the prediction accuracy was higher for stressed plants compared with control plants. This might have resulted from the imaging analysis process. Relatively small plants, stressed plants in this case, would gain more clear images due to less overlapping or less area out of range. Therefore, image quality would be an important variation source for our modeling and should be taken into consideration for any application.

## Discussion

Biomass is a complex but important trait in functional ecology and agronomy for studying plant growth, crop producing potential, and plant regeneration capabilities. Many different techniques, either destructive or nondestructive, have been used to estimate biomass [1–3, 5–17]. Compared with the traditional destructive methods for measuring biomass, nondestructive imaging methods provide a faster, more accurate approach for plant phenotyping. In recent years, more and more high-throughput plant phenotyping platforms have been set up and applied worldwide. Accordingly, it becomes a current challenge to establish models utilizing the big datasets gained from high-throughput imaging systems. Accurately predicting biomass from image data requires efficient mathematical models as well as representative image-derived features.

In this study, we have presented a systematic analysis of relationships between plant biomass accumulation and image-derived signals to confirm the assumption that biomass can be accurately predicted from image-based parameters. We built a random forest model of biomass accumulation using a comprehensive list of representative image-based features. The comparison between a random forest model and alternative regression models indicated that the RF model outperforms other models in terms of (1) better predictive power—especially in comparison with the linear model, confirming the complex phenotypic architecture of biomass, (2) better outperformance than a single feature prediction model—arguing the complex phenotypic makeup of biomass, and (3) feasible biological interpretability—the ability to readily extract information about the importance of each feature in prediction. The high prediction accuracy based on this model, in particular the cross-experiment performance, is promising to relieve the phenotyping bottleneck in biomass measurement in breeding applications. For example, based on the established small reference dataset that is used to train an RF model, it is possible to predict biomass in several large plant populations within 1 experiment or across several experiments using image data by taking advantage of high-throughput phenotyping technologies. Alternatively, the model can be trained from a much larger reference panel of plants that are grown in diverse environmental conditions, which is then applied to a diverse set of experiments. The first evidence for this notion is the observation that our model showed more predictive power in plants with 2 treatments than with a single treatment (Fig. 3, B and D). Indeed, when applying our model to the combined dataset from all 3 experiments, we found that the prediction accuracy remains very high (*R*^2^ = 0.96 and *r* = 0.98, average values from 10 times the 10-fold cross-validation). To keep the high prediction accuracy in other applications, there are some points that should be taken into consideration. Considering the environmental effects on biomass accumulation, the application of our model will require the testing experiments to show similar conducted conditions as those of the reference experiments. This means that the plant cultivation conditions should be standardized and any noise that might lower image quality should be avoided. Another approach to improve applicability of models, which could not be tested in this study, would be to improve the database for the training by acquiring data from additional environmental sensors. Temperature, humidity, and illumination data would certainly help to explain differences in the growth patterns among experiments, performed in different growth seasons. To this end, we expect that our approach is extensible by incorporating such sensor data in the data matrices. Furthermore, our results can provide suggestive hints for biologists to set up phenotyping infrastructures for investigation of plant biomass. For instance, a visible light imaging system would be sufficient to accurately predict fresh weight based on the observation that geometric features alone show high prediction accuracy (Fig. 4A). However, to investigate dry weight, it would be helpful to include an additional near-infrared camera system under normal growth conditions and an additional fluorescence camera system under drought stress conditions (Fig. 4B).

In contrast to previous studies [2, 3, 6, 7, 18, 32–37], in which biomass was investigated using only a single image-derived parameter (such as projected area) or several geometric parameters, our analyses extended these studies by incorporating more representative features that cover both structural and physiological-related properties into a more sophisticated model. Although the predictive power of our model is roughly higher than that of single feature-based prediction, such as digital volume (Fig. 3) [20], our model also reveals the relative contribution of individual features in the prediction of biomass. The information regarding the importance of each feature will offer new insights into the phenotypic determinants of plant biomass outcome. Interestingly, we found that several top ranked features, such as digital volume and NIR intensity, showed genetic correlations with biomass of fresh weight (Fig. 4C) [20], implying that these top-ranked features may represent the main “phenotypic components” of biomass outcome and that they can be further used to dissect genetic components underlying biomass accumulation. As image-based high-throughput phenotyping in plants developed mainly in recent years, and therefore few corresponding modeling studies have been performed, we believe that our model could be further improved when new types of cameras and/or newly defined features become available.

In summary, we have developed a quantitative model for dissecting the phenotypic components of biomass accumulation based on image data. Apart from predicting biomass outcome, the methods can be used to determine the most important image-based features related to plant biomass accumulation, which are promising for subsequent genetic mapping to uncover the genetic basis of biomass.

### Potential implications

As high-throughput plant phenotyping is a technique that is becoming more and more widely used for automated phenotype in plant research, especially in plant breeding, we anticipate that the methodologies proposed in this work will have various potential applications. We anticipate that the analysis results will be useful to advance our views of the phenotypic determinants of plant biomass outcome, and the statistical methods can be broadly used for other plant species and therefore assist plant breeding in the context of phenomics.

## Materials and Methods

### Germplasm and experiments

Barley plant image data were obtained as described previously [20, 30]. Briefly, a core set of 16 2-rowed spring barley cultivars (*Hordeum vulgare* L.) and 2 parental cultivars of a double haploid (DH) were monitored for vegetative biomass accumulation. Three independent experiments with identical setup were performed in a (semi-)controlled greenhouse at IPK by using the automated phenotyping and imaging platform LemnaTec-Scanalyzer 3D. Experiments were performed consecutively from May to November 2011 over a period of 58 days each (Supplemental Table S1). The greenhouse setup enabled sowing for the next experiment 2 days before the old experiment ended. For this, new pots were placed in the middle of the greenhouse, while the old experiment was still on the conveyer belts.

Each experiment consisted of 2 treatments: well-watered (control treatment) and water-limited (drought stress treatment). In each treatment, 9 plants per core set cultivar as well as 6 plants per DH parent were tested. This resulted in a total of 312 plants per experiment, corresponding to the maximal capacity of the phenotyping platform. Watering and imaging were performed daily. Drought stress was imposed by intercepting water supply from 27 days after sowing (DAS 27) to DAS 44. Stressed plants were rewatered at DAS 45. In total, for each experiment, about 100 GB of raw (image) data was accumulated. At the end of experiments (DAS 58), plants were harvested to measure above-ground biomass in the form of plant fresh weight (for all experiments) and/or dry weight (for experiment 1).

### Image analysis

Image datasets were processed by the barley analysis pipelines in the IAP software (version 1.1.2) [19]. Analyzed results were exported in the csv file format via IAP functionalities, which can be used for further data inspection. The result table includes columns for different phenotypic traits and rows as plants are imaged over time. The corresponding metadata are included in the resulting table as well.

Each plant was characterized by a set of phenotypic traits also referred to as features, which were grouped into 4 categories: geometric features, fluorescence-related features, color-related features, and near-infrared-related features. These traits were defined by considering image information from different cameras (visible light, fluorescence, and near infrared) and imaging views (side and top views). See the IAP online documentation [38] for details about trait definition.

### Feature selection

Feature selection was performed with the same procedure as described in Chen et al. [20]. We applied the feature selection technique to each dataset. Generally, we captured almost identical subset features from different datasets. We manually added several representative traits due to removal by variance inflation factors. For example, the digital volume and projected area are highly correlated with each other, but we kept both of them, because we would investigate the predictive power of both features. Moreover, the regression models we used are insensitive to collinear features. We thus kept as much of the representative features as possible. To apply the prediction models among different datasets, a common set of features supported by all the datasets was used.

### Data transformation

Each plant can be presented by a representative list of phenotypic traits, resulting in a matrix *X*_*n* × *m*_ for each experiment, where *n* is the number of plants and *m* is the number of phenotypic traits. Missing values were filled by mean values of other replicated plants. To make the image-derived parameters from diverse sources comparable, we normalized the columns of *X* by dividing the values with the maximum value of each column across all plants. Plants with empty values of manual measurements (FW and DW) were discarded for analysis. These transformed datasets were subjected to regression models.

### Hierarchical clustering analysis and PCA

Hierarchical clustering analysis and principle component analysis were performed on the transformed data matrix *X*_*n* × *m*_ in the same way as described in Chen et al. [20]. We also performed HCA using the genotype-level mean value of FW data to check the similarity of overall plant growth patterns in different experiments.

### Models for predicting plant biomass

To understand the underlying relationship between image-derived parameters and the accumulated biomass (such as FW and DW), we constructed predictive models based on 4 different machine-learning methods: MLR, MARS, RF, and SVR. In these models, the normalized phenotypic profile matrices *X*_*n* × *m*_ for a representative list of phenotypic traits were used as predictors (explanatory variables) and the measured DW/FW as the response variable *Y*.

All these models were implemented in R (release 2.15.2) [39]. To assess the relative contribution of each phenotypic trait to predicting the biomass, we also calculated the relative feature importance for each model. Specifically, for the MLR model, we used the “lm” function in the base installation packages. The relative importance of predictor variables in the MLR model was estimated by a heuristic method [40], which decomposes the proportionate contribution of each predictor variable to *R*^2^. For MARS, we used the “earth” function in the *earth* R package. The “number of subsets (nsubsets)” criterion (counting the number of model subsets that include the variable) was used to calculate the variables’ feature importance, which is implemented in the “evimp” function. For the RF model, we used the *randomForest* R package, which implements Breiman's random forest algorithm [31]. We chose the “%IncMSE” (increase of mean squared error) to represent the criteria of relative importance measure. For SVR, we utilized the *e1071* R package, which provides functionalities to use the *libsvm* library [41]. The absolute values of the coefficients of the normal vector to the “optimal” hyperplane can be considered the relative importance of each predictor variable contributing to regression [42, 43].

### Evaluation of the prediction models

To evaluate the performance of the predictive models, we adopted a 10-fold cross-validation strategy to check the prediction power of each regression model. Specifically, each dataset was randomly divided into a training set (90% of plants) and a testing set (10% of plants). We trained a model on the training data and then applied it to predict biomass for the testing data. Afterwards, the predicted biomass in the testing set was compared with the manually measured biomass. The predictive accuracy of the model can be measured by

1. the Pearson correlation coefficient (*r*) between the predicted values and the observed values;
2. the coefficient of determination (*R*^2^), which equals the fraction of variance of biomass explained by the model, defined as
  \begin{equation*}
{R^2} = 1 - \ \frac{{S{S\,_{res}}}}{{S{S\,_{tot}}}} = 1 - \frac{{\mathop \sum \nolimits_{i\ = \ 1}^n {{\left( {{y_i} - {{\hat{y}}_i}} \right)}^2}}}{{\mathop \sum \nolimits_{i\ = \ 1}^n {{\left( {{y_i} - \bar{y}} \right)}^2}}},\end{equation*}

where *SS* _*res*_ and *SS* _*tot*_ are the sum of squares for residuals and the total sum of squares, respectively, ${\hat{y}_i}$ is the predicted biomass of the *i*th plant, *y_i_* is the observed biomass of the *i*th plant, $\bar{y}$ is the mean value of the observed biomass, and

1. the root mean squared relative error of cross-validation is defined as
  \begin{equation*}{\rm{RMSRE}} = \sqrt {\frac{{\mathop \sum \nolimits_{i = 1}^s {{\left( {\frac{{{y_i} - {{\hat{y}}_i}}}{{{y_i}}}} \right)}^2}}}{s}} \ ,\end{equation*}

where *s* denotes the sample size of the testing dataset.

We repeated the cross-validation procedure 10 times. The mean and standard deviation of the resulting *R*^2^ and RMSRE values were calculated across runs.

To evaluate the applicability of our methods across seasons (thus different growth environments) and treatments (e.g., control vs drought stress) in the same season, we applied the models in different contexts with cohort validation. Specifically, we trained the biomass prediction models under 1 specific context and predicted biomass in another different context and vice versa. The predictive accuracy of the model was evaluated based on the measures *R*^2^ and RMSRE, as described above. Furthermore, the predictive power was reflected by the bias μ between the predicted and observed values, defined as

\begin{equation*}\mu \ = \frac{1}{n}\ \cdot \sum\nolimits_{i = 1}^n {\frac{{{{\hat{y}}_i} - {y_i}}}{{{y_i}}}} ,\end{equation*}

where *n* denotes the sample size of the dataset. This bias indicates over- (μ > 0) or underestimation (μ < 0) of biomass.

## Availability of source code and requirements

- Project name: Modeling of plant biomass accumulation with HTP data
- Project home page: https://github.com/htpmod/HTPmod
- Operating system(s): Windows, Linux, and Mac OS
- Programming language: R
- License: open source under GNU GPL v3.0

## Availability of supporting data and materials

The raw image datasets, as well as analyzed data supporting the results of this article, are available in the PGP repository [44] under https://dx.doi.org/10.5447/IPK/2017/24, https://dx.doi.org/10.5447/IPK/2017/25, and https://dx.doi.org/10.5447/IPK/2017/26, according to the ISA-Tab format and the recommendations of the Minimum Information About a Plant Phenotyping Experiment (MIAPPE) standard [45]. The selected data for modeling are available in Supplemental Data S1. Supporting data, including metadata tables, raw image files, and an archival copy of HTPmod are also available via the *GigaScience* repository, *Giga*DB [46].

### Additional files

Supplemental Figure S1. The relative importance of image-based features in the prediction of biomass in control plants. Refer to Fig. 4 for legend. The calculation was based on control plants.

Supplemental Figure S2. The relative importance of image-based features in the prediction of biomass in stressed plants. Refer to Fig. 4 for legend. The calculation was based on stressed plants.

Supplemental Table S1. Overview of 3 high-throughput phenotyping experiments in barley.

Supplemental Data S1. Manual data and image-derived data in the 3 experiments.

### Abbreviations

DAS: days after sowing; DW: dry weight; FLUO: fluorescence; FW: fresh weight; HCA: hierarchical clustering analysis; HTP: high-throughput phenotyping; MARS: multivariate adaptive regression splines; MLR: multivariate linear regression; NIR: near-infrared; PCA: principal component analysis; PCC: Pearson correlation coefficient; RF: random forest; RMSRE: root mean squared relative error; SVR: support vector regression.

### Competing interests

The authors declare that they have no competing interests.

### Funding

This work was supported by the Leibniz Institute of Plant Genetics and Crop Plant Research (IPK), the Robert Bosch Stiftung (32.5.8003.0116.0) and the Federal Agency for Agriculture and Food (BEL; 15/12-13, 530-06.01-BiKo CHN), and the Federal Ministry of Education and Research (BMBF; 0315958A and 031A053B). This research was furthermore enabled with support of the European Plant Phenotyping Network (EPPN; grant agreement No. 284 443), funded by the FP7 Research Infrastructures Programme of the European Union.

### Author contributions

D.C. designed the research. C.K. and M.C. supervised the project. K.N. and G.A. performed the LemnaTec experiments. D.A. created the ISA-Tab formatted description and uploaded data records in the PGP repository. J.M.P. and C.K. analyzed image data. D.C. implemented the methods, analyzed data, interpreted the results, and wrote the manuscript, with contributions from R.S. All authors read and approved the final version of the article.

## Supplementary Material

GIGA-D-17-00225_Original_Submission.pdf

GIGA-D-17-00225_Revision_1.pdf

GIGA-D-17-00225_Revision_2.pdf

Response_to_Reviewer_Comments_Original_Submission.pdf

Response_to_Reviewer_Comments_Revision_1.pdf

Reviewer_1_Report_(Original_Submission) -- Malia Gehan 25 Sep 2017 Reviewed

Reviewer_1_Report_(Revision_1) -- Malia Gehan 27 Nov 2017 Reviewed

Reviewer_2_Report_(Original_Submission) -- Christian Fournier 16 Oct 2017 Reviewed

Reviewer_2_Report_(Revision_1) -- Christian Fournier 04 Dec 2017 Reviewed

Supplemental material
