# Supplementary material for: Predicting plant biomass accumulation from image-derived parameters
Source: Gigascience. 2018 Jan 16;7(2):1–13. doi: 10.1093/gigascience/giy001 (PMC5827348; doi:10.1093/gigascience/giy001)
Supplement: Supplemental material [file giy001_supp.zip › Supplemental Figures.pdf]

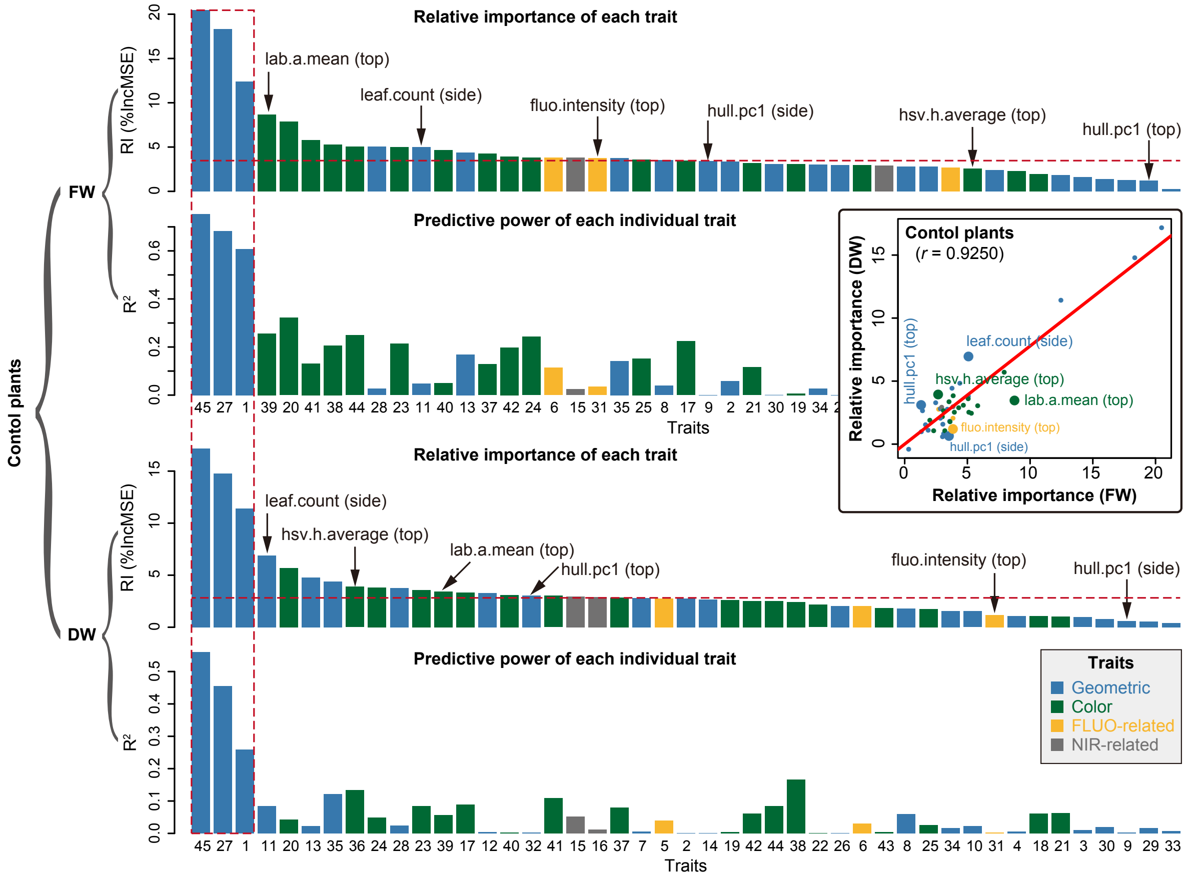


**Figure S1: The relative importance of image-based features in prediction of biomass in control plants.** Refer to Figure 4 for legend. The calculation is based on control plants.

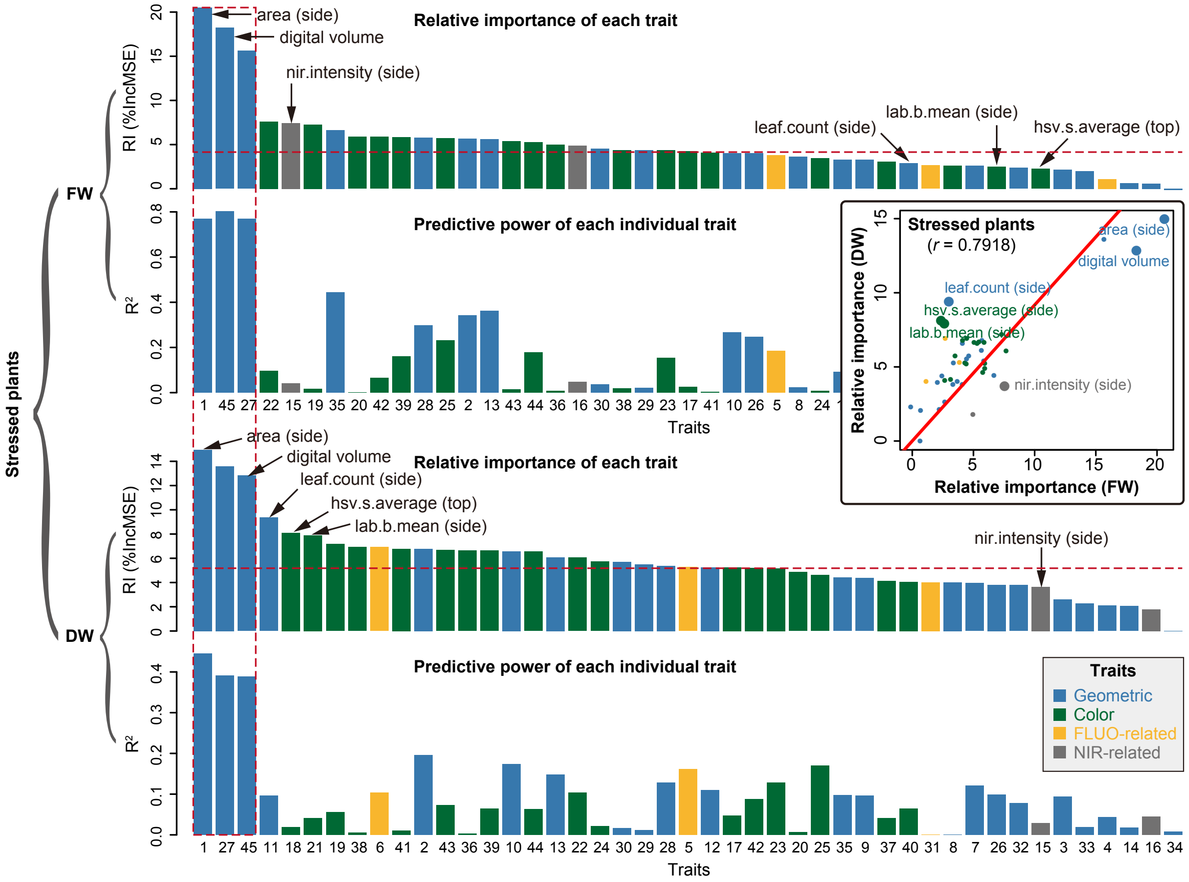


**Figure S2: The relative importance of image-based features in prediction of biomass in stressed plants.** Refer to Figure 4 for legend. The calculation is based on stressed plants.
