# Supplementary material for: Predicting plant biomass accumulation from image-derived parameters
Source: Gigascience. 2018 Jan 16;7(2):1–13. doi: 10.1093/gigascience/giy001 (PMC5827348; doi:10.1093/gigascience/giy001)
Supplement: Response_to_Reviewer_Comments_Original_Submission.pdf [file giy001_response_to_reviewer_comments_original_submission.pdf]

Author's Response To Reviewer Comments

We would like to thank both reviewers for their time to evaluate our manuscript and for their helpful comments/suggestions on our manuscript. It is our belief that this revised manuscript is significantly improved as a result of the changes suggested in the previous round of review. We explained point-by-point the changes made in response to the comments, and highlighted all the changes throughout the revised manuscript in blue. Our replies start with [Response].

Reviewer #1
Image datasets are available and are a valuable community resources. The code is available, which is great. While I definitely appreciate the authors work, I don't think the data support some of the statement throughout the paper, especially when it comes to the wording regarding MLR vs other models, unless further clarification can be provided (Figure 3). In some of the conditions (stress for example) MLR looks better than the other models. The inclusion of color, NIR, and Fluor traits into models is interesting.
[Response] We appreciate the reviewer's assessment of our work and his/her comments on our manuscript. We realized that some of the sentences in the manuscript might be overstated by reading the questions raised below. In the revised manuscript, we changed some parts and gave the statement more carefully (Response 1.7).

Lines 14-15: I think this statement needs to be qualified by saying that it is a challenge to find a predictive biomass model across experiments, not that it is a challenge to find a biomass model 'in the context of high-throughput phenotyping', which is vague and I don't think accurate without further clarification considering the number of previous papers that model biomass from images with high correlation to ground truth measurements.
[Response 1.1] We thank the reviewer for this valuable suggestion. We rewrote the statement according to the suggestion in the 'Abstract' section.

Lines 34 to 40: lacking in citations of literature. Introduction in general needs improvement in terms of the previous literature that it cites.
[Response 1.2] We thank the reviewer for pointing out this. We have added some new references and re-organized some text in the section 'Introduction' (line 36-44).

The second paragraph of the intro is a very limited short review of the literature but there are a number of papers that model biomass using ht-phenotyping that are not represented including Yang et al 2014 (nature communications), Montest et al. 2011 (Field Crops Research), Fahlgren et al. 2015 (Molecular Plant) to name a few.
[Response 1.3] We appreciate the reviewer's nice suggestion. We have added some related references in the revised manuscript (line 44-50).

Line 45: "On the other hand, to produce reliable assessments, suitable model types needs to be established and model construction requires integration of many components such as efficient mathematical analysis and representative data." Very vague.
Line 58: Please clarify this statement: "Another concern is that the number of traits used in these studies were quite limited and perhaps not representative enough. Therefore, a more effective and powerful model is needed to overcome these limitations and to allow better utilization of the

image-based plant features which are obtained from non-invasive phenotyping approaches." Not sure what this means exactly, very vague considering that the papers mentioned do have models of biomass that are not 'perfect' but do have high heritability and correlation with ground truth measurements.
[Response 1.4/1.5] We have rephrased related sentences in the second paragraph of the ‘Introduction’ section (line 62-65).

I think the authors need to adjust the justification of their research to stress that there needs to be biomass models that can be used across experiments/environment/treatments, which they do say, but needs to be stated more clearly. In general, many of the justification statements, which are pointed out in points 3 and 4 above are obscure to the point that they lose meaning.
[Response 1.6] We rephrased these statements in this revised version (line 69-73).

Line 146: "Although the performance of these models was roughly similar, RF, SVR and MARS methods had better performance than the MLR method for prediction of both FW (Fig. 3B) and DW (Fig. 3D), implying a nonlinear relationship between image-based phenotypic profiles and biomass output." This doesn't seem accurate, it looks like MLR has just as good predictive power in many of the situations presented. I don't think you can say that MLR and the others are roughly similar and then say that this implies a nonlinear relationship. Can this conclusion be clarified? It seems like there are only small differences between the models.
[Response 1.7] We thank the reviewer for cautioning us to avoid overstatements in our manuscript. We have revised the manuscript in the ‘Results’ section so as not to overstate our observations (line 153-165).

Regardless of whether or not random forest is the 'best' model, the data doesn't seem to support the statement that the RF model 'largely' outperformed the other models. This only seems accurate under the control condition, can this be clarified?
[Response 1.8] We agree with the reviewer that the statement wasn’t proper here. We have corrected this point in the revised manuscript.

Line 238: "Although previous attempts have been made to estimate plant biomass from image data, most of these studies consider only a single image-based feature or very few features in their models which are often linear-based, ignoring the fact that the phenotypic components underlying biomass accumulation are presumably complex. Accurately predicting biomass from image data requires efficient mathematical models as well as representative image-derived features." I disagree with the authors on this point, if biomass can be modeled with a few features with high correlation why does it matter if they presume that it is complex? Their more complex models were still decreased in R2 with environmental differences and between experiments and I don't find the data suggesting that RF model outperforming other models (particularly MLR) convincing without further clarification.
[Response 1.9] We agree with the reviewer’s comment that it would be good that if biomass can be modelled with a few features with high correlation. What we meant or afraid is that using too less feature might lead to under-estimated or over-estimated results since other features were not considered and evaluated. But nevertheless, we removed this sentence in this revised manuscript.

Reviewer #2:

The authors investigate the ability of deriving plant biomass (both fresh and dry mass) from 2D image-based features acquired with visible, fluorescent and NIR multi-view imaging systems operating on an automated high throughput phenotyping platform. In a first part, several multivariate statistical models are compared for their ability at predicting biomass for two treatments within a single experiment, on three independent datasets, detailed results being presented for one experiment. One of the best model, the random forest, is then further investigated for its capacity at making prediction across experiments, being trained on one experiment at a time or on one treatment of one experiment at a time. Finally, the relative importance of individual image-based traits in the prediction of either fresh or dry weight is presented for two treatments of one dataset.
Models and methods for model evaluation are clearly presented, and the overall quality of the text and Figure makes the paper easy to follow. The inclusion of other than visible images, the objective selection of image-based traits, the comparison of models and the use of 3 independent datasets clearly distinguish this paper from previous publications on the same subject. It provides the reader very valuable information on the current prediction capacity of the approach, together with a consistent methodology for analyzing other related practices.
[Response] We thank the reviewer for his/her assessment of our work and appreciate that the reviewer recognizes the advantages of our approaches and analysis.

However, I have two major concerns on the current version of this manuscript.
First, I think that some conclusions highlighted in the abstract or in the text are not completely in line (or at least sufficiently tempered) with what is demonstrated in the text or shown on the figures. In the abstract (line 19-20), it is highlighted that 'The results proved that plant biomass can be accurately predicted from image-based parameters using a random forest model'. To me this conclusion is clearly supported by data in the case of within experiment predictions, but not fully in the case of the cross experiment test (i.e. quite opposite to what is stressed line 21). My impression, given results presented Figure 5, is that in one case out of two, a model trained on one experiment alone could not accurately (or at least with not the same accuracy) predict the biomass, despite a repeated protocol. This result is per se very interesting, as it demonstrates an important limitation of the approach. It can however not be summarized by what is written line 19-21, 201-202, 209-210 or 253-257. On another occasion (line 148 and line 248), I found the conclusion ('the RF model largely outperformed other models') a bit exaggerated, as, on Figure 3, depending on the criteria, RF model performs very similar to MARS model for example.
[Response 2.1] We thank the reviewer for raising these points. 1) we agree with the reviewer that the prediction accuracy of the model across experiments is still lower in some case (mostly due to growth conditions changing over seasons). We changed relevant description in the text and added some text to discuss this point. 2) We agree with the reviewer on this point. We clarified this point in the revised version (see line 153-165).

Second, I did not manage to test the models, nor to reproduce the analysis with the provided data and source code. Concerning the data, image traits are provided for all experiments, but manual measurement on Dry Weight are missing. Concerning the code, the R-script provided does not fit to the provided dataset, thus making it difficult to test. More important, model code runs with errors at runtime ('not defined' errors). I also think, but this is only a suggestion, that, in addition to raw image files, providing binary masks of plants, that are of high importance for all traits analyzed here, could improve the re-use of this nice dataset.

[Response 2.2] We thank the viewer for raising this point. We now provided a separated R script 'run.R'to test the models. The test data used in our analysis were now deposited in the Github repository (https://github.com/htpmod/HTPmod). We also thank for the reviewer for this nice suggestion. Unfortunately, the files for the binary masks of plants were not kept anymore due to storage limitation. But the report files (generated by the IAP software) that include all trait values are still kept. We uploaded these report files alongside the raw image datasets for re-use of these data (see the section 'Availability of supporting data and materials').

Other minor points or comments for specific parts of the texts are provided bellow:
Line 72-74: I think this sentence would be better be placed in the Potential application section
[Response 2.3] We have moved the sentence to the section 'Potential Implications'.

Line 85: Do you mean that some image traits are more sensitive to physiological traits? I do not see why Fig 1B is illustrative for this point.
[Response 2.4] The reviewer is correct that some image traits are more sensitive to physiological traits. We agree with the reviewer that the citation of Fig. 1B is not proper here. We corrected this accordingly.

Line 98: In the context of phenotyping, it might also be useful to add Spearman rank correlation to the assessment
[Response 2.5] We agree with the reviewer's opinion. In principle, the assessment based on these two kinds of correlation coefficients is similar for good models. We prefer to choose Pearson correlation coefficient based on the assumption that the relationship of observed values and predicted values is linear.

Line 108: Fig 1B is only a heatmap image. May be a list of traits should be provided, or a reference to the supplementary data should be added here.
[Response 2.6] We want to thank the reviewer for bringing up this point. We added a reference to the Supplementary Data S1.

Line 117: Figure 2B is poorly informative as traits are not identified. This figure is also not commented in the text, I suggest removing it.
[Response 2.7] We agree to the reviewer's suggestion and removed Figure 2B in this revised version.

Line 144: I would find useful to make here perfectly clear that all the models were trained on the control + stress plants, to avoid any confusion with the 'cross treatment test' later on (Figure 6)
[Response 2.8] We thank the reviewer for pointing this out. We have corrected this in the revised manuscript.

Line 146-151: I found the analysis a bit confusing as, in the details, the ranking of the different methods varies, and I do not clearly see why RF 'largely outperforms' other methods (especially MARS).
[Response 2.9] We agree with the reviewer that this statement is overstated. We have corrected this issue in the revised manuscript (see line 153-165).

Line 152-155: The comparison with the widely used 'single feature' method is very interesting. Can you consider to add its score/line on the R2 and RMSRE?
[Response 2.10] This is indeed a good suggestion. We now added this value in Figure 3B and D.

Line 178: May be it is also worth noting in the text that geometric + color traits trust 13 out of 15 (FW) and 15 out of 15 (DW) first places, as these two types of data are widely available among phenotyping platform and yet not so often used in biomass predictions.
[Response 2.11] We appreciate the reviewer for this valuable comment. We have included this point in the revised manuscript.

Line 201 - 211: The text seems to me a bit too optimistic regarding the cross experiment predictions. Exp3 clearly shows a non-conservation of the relationship obtained in Exp1 or 2, and a clear loss of predictive power compared to within experiment training.
[Response 2.12] We admit that we are a bit overstate here. We have changed it in the revised version.

Line 281: typo: sophisticated
[Response 2.13] We appreciate the reviewer for pointing out this mistake. We have corrected this word in the revised manuscript.

Line 349: could you give an idea of the amount of such filled missing values?
[Response 2.14] After the feature selection step (e.g., outlier detection, reproducibility analysis and redundancy removal), the missing values for the remaining features are quite rare (much less than 1%).

Line 400: the formulation is a bit strange as it sounds like a conclusion already.
[Response 2.15] We have improved this sentence in the revised version.

Line 426: DW data are missing.
[Response 2.16] We have added DW data in the Supplemental Data S1.

Line 535: legend of figure 5 did not really apply to these figures. A complete legend should be added.
[Response 2.17] A new legend was added for Figure 5.
