# Supplementary material for: Predicting plant biomass accumulation from image-derived parameters
Source: Gigascience. 2018 Jan 16;7(2):1–13. doi: 10.1093/gigascience/giy001 (PMC5827348; doi:10.1093/gigascience/giy001)
Supplement: Response_to_Reviewer_Comments_Revision_1.pdf [file giy001_response_to_reviewer_comments_revision_1.pdf]

Author's Response To Reviewer Comments

We would like to thank both reviewers again for their further comments/suggestions to improve our manuscript. Our replies start with [Response].

Reviewer #1: Chen et al, appear to have addressed each reviewer comment, below are some minor language changes for the revised sections.
Minor changes (language changes)
1. Line 47: remove "some other traits" seems unnecessary
2. Line 64: change "they" to Buesmeyer et al. 2013, and change "make it a question" to "question"
3. Line 73 change besides to "Further"
4. Line 75 change to "due to a lack of datasets for assessment"
[Response] We appreciate the reviewer for pointing out these. We corrected these in the revised manuscript.

Reviewer #2: I thank the authors for the work done on the new manuscript and on Github, that address most of the concerns I raised in my first review.
The pipeline published on GitHub now works nicely and allows to reproduces the different analyses. I only had to install manually two packages (earth and e1071). They could be easily added to the list of dependency in the R script to completely automatize the installation.
[Response] We thank the reviewer for this suggestion. We fixed this issue in the 'run.R' script.

The authors also clarify their analysis of the comparison of models, and the overstatement concerning the RF model has been corrected.
I however still think that the abstract should be amended to better match the conclusions of the cross experiment test. The author acknowledged, in their response and in the text (line 226) that one cross experiment test leads to a loss of predictive accuracy.
It seems also obvious, from Figure 5, and this should probably be added to the text, that this loss of accuracy is not linked to a greater random dispersion of the points, but to a systematic model bias. I agree with the authors that this may be due to some changes in the experimental conditions. My point is that these changes are not completely captured by the model, even with the inclusion of non structural traits. I therefore still think that there is some overstatement/ambiguity in the abstract, in particular in the sentence' The high prediction accuracy based on this model, in particular the cross experiment performance, will contribute to relieve the phenotyping bottleneck in biomass measurement in breeding applications' .
This may however be easily fixed.
[Response] According to the reviewer's comment, we changed the sentence 'The high prediction accuracy based on this model, in particular the cross-experiment performance, will contribute to relieve the phenotyping bottleneck in biomass measurement in breeding applications.' to 'The high prediction accuracy based on this model will contribute to relieve the phenotyping bottleneck in biomass measurement in breeding applications. The prediction performance is still relatively high cross experiments under similar conditions.'
