# Supplementary material for: Predicting plant biomass accumulation from image-derived parameters
Source: Gigascience. 2018 Jan 16;7(2):1–13. doi: 10.1093/gigascience/giy001 (PMC5827348; doi:10.1093/gigascience/giy001)
Supplement: Reviewer_1_Report_(Original_Submission) -- Malia Gehan — 25 Sep 2017 Reviewed [file giy001_reviewer_1_report_(original_submission).pdf]

Reviewer Report

Title: Predicting plant biomass accumulation from image-derived parameters

Version: Original Submission **Date:** 25 Sep 2017

Reviewer name: Malia Gehan

Reviewer Comments to Author:

Image datasets are available and are a valuable community resources. The code is available, which is great. While I definitely appreciate the authors work, I don't think the data support some of the statement throughout the paper, especially when it comes to the wording regarding MLR vs other models, unless further clarification can be provided (Figure 3). In some of the conditions (stress for example) MLR looks better than the other models. The inclusion of color, NIR, and Fluor traits into models is interesting.

Lines 14-15: I think this statement needs to be qualified by saying that it is a challenge to find a predictive biomass model across experiments, not that it is a challenge to find a biomass model 'in the context of high-throughput phenotyping', which is vague and I don't think accurate without further clarification considering the number of previous papers that model biomass from images with high correlation to ground truth measurements.

Lines 34 to 40: lacking in citations of literature. Introduction in general needs improvement in terms of the previous literature that it cites.

The second paragraph of the intro is a very limited short review of the literature but there are a number of papers that model biomass using ht-phenotyping that are not represented including Yang et al 2014 (nature communications), Montest et al. 2011 (Field Crops Research), Fahlgren et al. 2015 (Molecular Plant) to name a few.

Line 45: "On the other hand, to produce reliable assessments, suitable model types needs to be established and model construction requires integration of many components such as efficient mathematical analysis and representative data." Very vague.

Line 58: Please clarify this statement: "Another concern is that the number of traits used in these studies were quite limited and perhaps not representative enough. Therefore, a more effective and powerful model is needed to overcome these limitations and to allow better utilization of the image-based plant features which are obtained from non-invasive phenotyping approaches." Not sure what this means exactly, very vague considering that the papers mentioned do have models of biomass that are not 'perfect' but do have high heritability and correlation with ground truth measurements.

I think the authors need to adjust the justification of their research to stress that there needs to be

biomass models that can be used across experiments/environment/treatments, which they do say, but needs to be stated more clearly. In general, many of the justification statements, which are pointed out in points 3 and 4 above are obscure to the point that they lose meaning.

Line 146 : "Although the performance of these models was roughly similar, RF, SVR and MARS methods had better performance than the MLR method for prediction of both FW (Fig. 3B) and DW (Fig. 3D), implying a nonlinear relationship between image-based phenotypic profiles and biomass output." This doesn't seem accurate, it looks like MLR has just as good predictive power in many of the situations presented. I don't think you can say that MLR and the others are roughly similar and then say that this implies a nonlinear relationship. Can this conclusion be clarified? It seems like there are only small differences between the models.

Regardless of whether or not random forest is the 'best' model, the data doesn't seem to support the statement that the RF model 'largely' outperformed the other models. This only seems accurate under the control condition, can this be clarified?

Line 238: "Although previous attempts have been made to estimate plant biomass from image data, most of these studies consider only a single image-based feature or very few features in their models which are often linear-based, ignoring the fact that the phenotypic components underlying biomass accumulation are presumably complex. Accurately predicting biomass from image data requires efficient mathematical models as well as representative image-derived features." I disagree with the authors on this point, if biomass can be modeled with a few features with high correlation why does it matter if they presume that it is complex? Their more complex models were still decreased in R2 with environmental differences and between experiments and I don't find the data suggesting that RF model outperforming other models (particularly MLR) convincing without further clarification.

Methods

Are the methods appropriate to the aims of the study, are they well described, and are necessary controls included? Yes

Conclusions

Are the conclusions adequately supported by the data shown? No

Reporting Standards

Does the manuscript adhere to the journal's guidelines on [minimum standards of reporting?]() Yes

Statistics

Are you able to assess all statistics in the manuscript, including the appropriateness of statistical tests used? Yes, and I have assessed the statistics in my report.

Quality of Written English

Please indicate the quality of language in the manuscript: Acceptable

Declaration of Competing Interests

Please complete a declaration of competing interests, considering the following questions:

- Have you in the past five years received reimbursements, fees, funding, or salary from an organisation that may in any way gain or lose financially from the publication of this manuscript, either now or in the future?
- Do you hold any stocks or shares in an organisation that may in any way gain or lose financially from the publication of this manuscript, either now or in the future?
- Do you hold or are you currently applying for any patents relating to the content of the manuscript?
- Have you received reimbursements, fees, funding, or salary from an organization that holds or has applied for patents relating to the content of the manuscript?
- Do you have any other financial competing interests?
- Do you have any non-financial competing interests in relation to this paper?

If you can answer no to all of the above, write 'I declare that I have no competing interests' below. If your reply is yes to any, please give details below.

I declare I have no competing interests.

I agree to the open peer review policy of the journal. I understand that my name will be included on my report to the authors and, if the manuscript is accepted for publication, my named report including any attachments I upload will be posted on the website along with the authors' responses. I agree for my report to be made available under an Open Access Creative Commons CC-BY license (http://creativecommons.org/licenses/by/4.0/). I understand that any comments which I do not wish to be included in my named report can be included as confidential comments to the editors, which will not be published.

I agree to the open peer review policy of the journal

To further support our reviewers, we have joined with Publons, where you can gain additional credit to further highlight your hard work (see: https://publons.com/journal/530/gigascience). On publication of this paper, your review will be automatically added to Publons, you can then choose whether or not to claim your Publons credit. I understand this statement.

Yes
