# Supplementary material for: Predicting plant biomass accumulation from image-derived parameters
Source: Gigascience. 2018 Jan 16;7(2):1–13. doi: 10.1093/gigascience/giy001 (PMC5827348; doi:10.1093/gigascience/giy001)
Supplement: Reviewer_2_Report_(Original_Submission) -- Christian Fournier — 16 Oct 2017 Reviewed [file giy001_reviewer_2_report_(original_submission).pdf]

Reviewer Report

Title: Predicting plant biomass accumulation from image-derived parameters

Version: Original Submission **Date:** 16 Oct 2017

Reviewer name: Christian Fournier

Reviewer Comments to Author:

The authors investigate the ability of deriving plant biomass (both fresh and dry mass) from 2D image-based features acquired with visible, fluorescent and NIR multi-view imaging systems operating on an automated high throughput phenotyping platform. In a first part, several multivariate statistical models are compared for their ability at predicting biomass for two treatments within a single experiment, on three independent datasets, detailed results being presented for one experiment. One of the best model, the random forest, is then further investigated for its capacity at making prediction across experiments, being trained on one experiment at a time or on one treatment of one experiment at a time. Finally, the relative importance of individual image-based traits in the prediction of either fresh or dry weight is presented for two treatments of one dataset.

Models and methods for model evaluation are clearly presented, and the overall quality of the text and Figure makes the paper easy to follow. The inclusion of other than visible images, the objective selection of image-based traits, the comparison of models and the use of 3 independent datasets clearly distinguish this paper from previous publications on the same subject. It provides the reader very valuable information on the current prediction capacity of the approach, together with a consistent methodology for analyzing other related practices.

However, I have two major concerns on the current version of this manuscript.

First, I think that some conclusions highlighted in the abstract or in the text are not completely in line (or at least sufficiently tempered) with what is demonstrated in the text or shown on the figures. In the abstract (line 19-20), it is highlighted that 'The results proved that plant biomass can be accurately predicted from image-based parameters using a random forest model'. To me this conclusion is clearly supported by data in the case of within experiment predictions, but not fully in the case of the cross experiment test (i.e. quite opposite to what is stressed line 21). My impression, given results presented Figure 5, is that in one case out of two, a model trained on one experiment alone could not accurately (or at least with not the same accuracy) predict the biomass, despite a repeated protocol. This result is per se very interesting, as it demonstrates an important limitation of the approach. It can however not be summarized by what is written line 19-21, 201-202, 209-210 or 253-257. On another occasion (line 148 and line 248), I found the conclusion ('the RF model largely outperformed other models') a bit exaggerated, as, on Figure 3, depending on the criteria, RF model performs very similar to MARS model for example.

Second, I did not manage to test the models, nor to reproduce the analysis with the provided data and source code. Concerning the data, image traits are provided for all experiments, but manual measurement on Dry Weight are missing. Concerning the code, the R-script provided does not fit to the provided dataset, thus making it difficult to test. More important, model code runs with errors at

runtime ('not defined' errors). I also think, but this is only a suggestion, that, in addition to raw image files, providing binary masks of plants, that are of high importance for all traits analyzed here, could improve the re-use of this nice dataset,.

Other minor points or comments for specific parts of the texts are provided bellow:

Line 72-74: I think this sentence would be better be placed in the Potential application section

Line 85: Do you mean that some image traits are more sensitive to physiological traits ? I do not see why Fig 1B is illustrative for this point.

Line 98: In the context of phenotyping, it might also be useful to add Spearman rank correlation to the assessment

Line 108: Fig 1B is only a heatmap image. May be a list of traits should be provided, or a reference to the supplementary data should be added here.

Line 117: Figure 2B is poorly informative as traits are not identified. This figure is also not commented in the text, I suggest removing it.

Line 144: I would find useful to make here perfectly clear that all the models were trained on the control + stress plants, to avoid any confusion with the 'cross treatment test' later on (Figure 6)

Line 146-151: I found the analysis a bit confusing as, in the details, the ranking of the different methods varies, and I do not clearly see why RF 'largely outperforms' other methods (especially MARS).

Line 152-155: The comparison with the widely used 'single feature' method is very interesting. Can you consider to add its score/line on the R2 and RMSRE ?

Line 178: May be it is also worth noting in the text that geometric + color traits trust 13 out of 15 (FW) and 15 out of 15 (DW) first places, as these two types of data are widely available among phenotyping platform and yet not so often used in biomass predictions.

Line 201 - 211: The text seems to me a bit too optimistic regarding the cross experiment predictions. Exp3 clearly shows a non-conservation of the relationship obtained in Exp1 or 2, and a clear loss of predictive power compared to within experiment training.

Line 281: typo: sophisticated

Line 349: could you give an idea of the amount of such filled missing values?

Line 400: the formulation is a bit strange as it sounds like a conclusion already.

Line 426: DW data are missing.

Line 535: legend of figure 5 did not really apply to these figures. A complete legend should be added.

Methods

Are the methods appropriate to the aims of the study, are they well described, and are necessary controls included? Yes

Conclusions

Are the conclusions adequately supported by the data shown? No

Reporting Standards

Does the manuscript adhere to the journal's guidelines on minimum standards of reporting? No

Statistics

Are you able to assess all statistics in the manuscript, including the appropriateness of statistical tests used? Yes, and I have assessed the statistics in my report.

Quality of Written English

Please indicate the quality of language in the manuscript: Acceptable

Declaration of Competing Interests

Please complete a declaration of competing interests, considering the following questions:

- Have you in the past five years received reimbursements, fees, funding, or salary from an organisation that may in any way gain or lose financially from the publication of this manuscript, either now or in the future?
- Do you hold any stocks or shares in an organisation that may in any way gain or lose financially from the publication of this manuscript, either now or in the future?
- Do you hold or are you currently applying for any patents relating to the content of the manuscript?
- Have you received reimbursements, fees, funding, or salary from an organization that holds or has applied for patents relating to the content of the manuscript?
- Do you have any other financial competing interests?
- Do you have any non-financial competing interests in relation to this paper?

If you can answer no to all of the above, write 'I declare that I have no competing interests' below. If your reply is yes to any, please give details below.

I declare that I have no competing interests

I agree to the open peer review policy of the journal. I understand that my name will be included on my report to the authors and, if the manuscript is accepted for publication, my named report including any attachments I upload will be posted on the website along with the authors' responses. I agree for my report to be made available under an Open Access Creative Commons CC-BY license (http://creativecommons.org/licenses/by/4.0/). I understand that any comments which I do not wish to be included in my named report can be included as confidential comments to the editors, which will not be published.

I agree to the open peer review policy of the journal

To further support our reviewers, we have joined with Publons, where you can gain additional credit to further highlight your hard work (see: https://publons.com/journal/530/gigascience). On publication of this paper, your review will be automatically added to Publons, you can then choose whether or not to claim your Publons credit. I understand this statement.

Yes
